# Proteomic profiling of striatal tissue of a rat model of Parkinson's disease after implantation of collagen‐encapsulated human umbilical cord mesenchymal stem cells

**DOI:** 10.1002/term.3081

**Published:** 2020-06-26

**Authors:** Anna Santaella, Hans J.C.T. Wessels, Purva Kulkarni, Jolein Gloerich, Bea Kuiperij, Bastiaan R. Bloem, Alain J. van Gool, Silvia Cabré, Verónica Alamilla, Marcel M. Verbeek

**Affiliations:** ^1^ Departments of Neurology and Laboratory Medicine Radboud University Medical Center Nijmegen The Netherlands; ^2^ Laboratory Medicine Radboud University Medical Center Nijmegen The Netherlands; ^3^ Center of Expertise for Parkinson & Movement Disorders, Donders Institute for Brain, Cognition and Behavior Radboud University Medical Center Nijmegen The Netherlands; ^4^ Pharmacology & Therapeutics and CÚRAM Centre for Research in Medical Devices National University of Ireland Galway Ireland; ^5^ CÚRAM Centre for Research in Medical Devices National University of Ireland Galway Ireland

**Keywords:** collagen hydrogels, mesenchymal stem cells, Parkinson's disease, proteomics, rat model, regenerative medicine

## Abstract

Parkinson's disease (PD) is the most common neurodegenerative disorder of movement worldwide. To date, only symptomatic treatments are available. Implantation of collagen‐encapsulated human umbilical cord mesenchymal stem cells (hUC‐MSCs) is being developed as a novel therapeutic approach to potentially modify PD progression. However, implanted collagen scaffolds may induce a host tissue response. To gain insight into such response, hUC‐MSCs were encapsulated into collagen hydrogels and implanted into the striatum of hemi‐Parkinsonian male Sprague–Dawley rats. One or 14 days after implantation, the area of interest was dissected using a cryostat. Total protein extracts were subjected to tryptic digestion and subsequent LC–MS/MS analyses for protein expression profiling. Univariate and multivariate analyses were performed to identify differentially expressed protein profiles with subsequent gene ontology and pathway analysis for biological interpretation of the data; 2,219 proteins were identified by MaxQuant at 1% false discovery rate. A high correlation of label‐free quantification (LFQ) protein values between biological replicates (*r* = .95) was observed. No significant differences were observed between brains treated with encapsulated hUC‐MSCs compared to appropriate controls. Proteomic data were highly robust and reproducible, indicating the suitability of this approach to map differential protein expression caused by the implants. The lack of differences between conditions suggests that the effects of implantation may be minimal. Alternatively, effects may only have been focal and/or could have been masked by nonrelevant high‐abundant proteins. For follow‐up assessment of local changes, a more accurate dissection technique, such as laser micro dissection, and analysis method are recommended.

## 1 INTRODUCTION

Parkinson's disease (PD) is the second most common neurodegenerative disease worldwide affecting 1% of people older than age 60 (Kalia & Lang, 2015). Neural degeneration is associated with the presence of Lewy bodies, which contain aggregates of the protein α‐synuclein. The prodromal phase of PD features several non‐motor symptoms that persist in later stages. Only in later stages the typical motor symptoms appear because, by then, 50%–80% of dopaminergic neurons have died (Braak et al., 2003). Motor symptoms include rigidity, bradykinesia, and resting tremor (Kalia & Lang, 2015). Current treatments for PD are symptomatic and target the dopaminergic system using pharmacotherapy (mainly L‐DOPA) or, in later stages of the disease, deep brain stimulation. However, there is yet no disease‐modifying drug (Lindholm et al., 2016).

Cell therapy emerged as a promising therapeutic treatment for neurodegenerative diseases such as PD (Venkataramana et al., 2010; Venkatesh & Sen, 2017; Yasuhara, Kameda, Sasaki, Tajiri, & Date, 2017). Implanted cells could reinnervate and recover functionality of the disrupted neuronal network and/or release trophic factors and anti‐inflammatory proteins to promote neuronal survival. It has been reviewed that preclinical and clinical studies showed controversial effects after cell implantation. Whereas some studies showed substantial and long‐lasting functional benefits, others showed marginal or no effects and dyskinesia as an undesired side effect (Towns, 2017; Winkler, Kirik, & Bjorklund, 2005). Several preclinical studies also reported poor cell survival and poor engraftment after implantation and, in some cases, migration of the implanted cells to other areas of the brain (Lee, Choi, Cha, & Hwang, 2015; Steward, Sharp, & Matsudaira Yee, 2014). Biomaterials appear to be a good solution to support cells during the first days after implantation, to protect them from the immune system, and to narrow their effect to the area of interest. The biomaterial of choice is not a trivial decision. Biomaterials for brain regeneration need to be biodegradable, non‐immunogenic, have a softness similar to that of the brain tissue, and control cell behavior, such as adhesion, migration, apoptosis, proliferation, and differentiation (Sengupta & Heilshorn, 2010; Zhu & Marchant, 2011). Polyethylene glycol (PEG)‐crosslinked collagen scaffolds are good candidates because of their biocompatibility and ability to provide a three‐dimensional structure that mimics the natural extracellular matrix (Zhu & Marchant, 2011). As important as the biomaterial itself is the selected type of cells. Human umbilical cord mesenchymal stem cells (hUC‐MSCs) are birth‐associated stem cells that present unique advantages for use in regenerative medicine. hUC‐MSCs are obtained through non‐invasive procedures, rise fewer ethical concerns because of their non‐embryonic origin, and they are highly available. Moreover, hUC‐MSCs, in comparison with non‐birth‐associated stem cells, present higher proliferative capacity, higher life span, higher differentiation potential, do not induce teratomas, harbor strong immunomodulatory capacities, and express genes involved in neuronal development (Castro‐Manrreza & Montesinos, 2015; El Omar et al., 2014; Hass, Kasper, Bohm, & Jacobs, 2011). Noteworthy, they do not express HLA‐DR receptors and have a low expression level of HLA‐ABC, suggesting that these cells could be good candidates for allogeneic cell therapy (El Omar et al., 2014).

To accurately assess the efficacy and safety of collagen‐encapsulated hUC‐MSCs as a therapy for PD, it is important to investigate the host tissue response following implantation. To our knowledge, past studies on cell therapy mainly focused on analyzing specific markers of inflammation (microgliosis and astrogliosis), cell survival, and cell reinnervation (Hoban et al., 2013; Moriarty, Cabre, Alamilla, Pandit, & Dowd, 2019; Moriarty, Pandit, & Dowd, 2017). We considered it important to deepen the knowledge of host tissue response, in which we focused on proteins as the active mediators of biological response. We addressed this using shotgun proteomics, a mass spectrometry‐based approach that allows for large‐scale analysis of proteins in a given tissue at a given state. Thus, we aimed to investigate the host tissue response at a proteomic level of the 6‐hydroxydopamine (6‐OHDA) rat model of PD following implantation of collagen‐encapsulated hUC‐MSCs. We hypothesized that 24 h after implantation, markers of gliosis and inflammation could be identified, but that they would normalize over time and then neurotrophic and metabolic changes would occur. We aimed to obtain novel insights into therapeutic mechanisms of collagen‐encapsulated hUC‐MSCs, which will allow us to establish changes needed in the collagen hydrogel composition to improve its safety and therapeutic efficacy.

## 2 METHODS

### 2.1 Animals

All experiments involving the use of animals were carried out in the National University of Ireland, Galway, in accordance with relevant guidelines and regulations, completed under license by the Irish Department of Health and Children and the Irish Health Products Regulatory Authority, performed in compliance with the European Union Directive 2010/63/EU and S. I No. 543 of 2012, and approved by the Animal Care and Research Ethics Committee at the National University of Ireland, Galway. Male Sprague–Dawley rats, weighing 200–225 g on arrival, were obtained from Charles River, UK. Animals (*n* = 40) were housed in groups of four per cage, on a 12/12‐h light/dark cycle, at 19–23 °C, with relative humidity levels maintained between 40% and 70%. For the duration of the experiment, animals were allowed food and water *ad libitum.*

### 2.2 Fabrication of cross‐linked Type I bovine collagen hydrogels and hUC‐MSCs encapsulation

Collagen hydrogel production was performed as previously described elsewhere (Moriarty et al., 2017; Moriarty et al., 2019). Briefly, all components were maintained on ice to prevent gelation. For a final volume of 200 μl, 133.4 μl of 2‐mg/ml Type I collagen (Collagen Solutions, Galway, Ireland) was mixed with 10 μl of 10× phosphate buffered saline (PBS) and neutralized with 1.6 μl of 1‐M NaOH to reach a pH 7. Then 12.5 μl of poly (ethylene glycol) ether tetrasuccinimidyl glutarate (4S‐StarPEG; 4 mg/ml; JenKem Technology, Plano, Texas, USA) diluted in 1× PBS was added, and the solution was thoroughly mixed. Finally, 10 μl of hUC‐MSCs (Orbsen Therapeutics, Galway, Ireland; Passage 3) suspension was added to the collagen/PBS/cross‐linker solution at a final concentration of 10,000 cells/μl for in vivo studies and also 2,500 cells/μl for in vitro studies. The cell‐seeded collagen hydrogel was maintained on ice prior to transplantation to prevent gelation.

### 2.3 Human umbilical cord mesenchymal stem cells in vitro analyses

hUC‐MSCs were purchased at Orbsen Therapeutics at passage number 2. hUC‐MSCs were cultured in a 6‐well plate under hypoxic conditions (2% O_2_, 5% CO_2_) at 37 °C with minimum essential medium alpha (α‐MEM; Thermo Fisher Scientific, USA) containing 10% of fetal bovine serum (Sigma), 1% penicillin streptomycin (Biosciences), and 1 ng/ml of human fibroblast growth factor (hFGF). Media were changed every other day. At passage number 3, cells were harvested to proceed with collagen encapsulation. Cells were encapsulated at either 10,000 or 2,500 hUC‐MSCs/μl into 3 μl of collagen hydrogel as described above. After 5 days, collagen hydrogels were degraded for 30 min at 37 °C with collagenases from *Clostridium histolyticum* (C2674‐100MG, Sigma‐Aldrich; 0.5‐mg collagenases/ml of 1× PBS containing CaCl_2_). Cell viability was assessed by using the trypan blue exclusion test.

### 2.4 6‐OHDA lesions and transplantation

**FIGURE 1:**
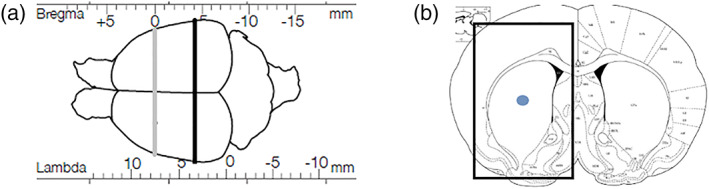
Injection area. (a) Stereotaxic coordinates. In black, lesion in the medial forebrain bundle using 6‐hydroxydopamine (AP: −4.0‐mm bregma). In grey, transplantation plane of collagen‐encapsulated cells in the striatum (AP: 0.0‐mm bregma). Both lesion and transplantation were performed unilaterally at the right hemisphere. (b) Coronal view of the transplantation side. The dot represents the implant and the square the dissected area. AP, anterior–posterior [Colour figure can be viewed at wileyonlinelibrary.com]

All surgeries were performed under isoflurane anesthesia (5% in O_2_ for induction and 2% in O_2_ for maintenance) in a stereotaxic frame with the nose bar set at −4.5 mm (intramedial forebrain bundle [MFB]) or −2.3 mm (intrastriatal). To induce Parkinsonism, rats received a unilateral MFB lesion using 6‐OHDA (12 μg in 3‐μl 0.1% ascorbate saline) at stereotaxic coordinates anterior–posterior (AP) −4.0 mm, medial–lateral (ML) −1.3 mm (from bregma), and dorsal–ventral (DV) −7.0 mm below dura. For transplant surgery, rats received unilateral intrastriatal transplants in a volume of 3 μl of hUC‐MSCs alone (10,000 cells/μl; *n* = 10), collagen alone (*n* = 10), collagen‐encapsulated hUC‐MSCs (10,000 cells/μl; *n* = 10) or saline solution (*n* = 10) at stereotaxic coordinates AP 0.0, ML −3.7 mm (from bregma), and DV −5.0 mm below dura (Figure 1). Injection speed rate was set at 1 μl/min, and the needle remained *in situ* for another 2 min to allow for diffusion. Animals were sacrificed 1 day (*n* = 20) or 14 days (*n* = 20) after implantation by terminal anesthesia and were transcardially transfused with 100‐ml heparinized saline solution. Brains were rapidly removed, snap frozen in liquid nitrogen, and stored at −80 °C until further analysis.

### 2.5 Sample preparation for mass spectrometry analysis

Coronal sections of the right striatum containing the implants, the same area of the intact left hemisphere and the 6‐OHDA‐lesioned area of the right hemisphere, were cut at a thickness of 100 μm in a cryostat (Microm HM550; Thermo Fisher Scientific) and ground in 8‐M urea in 10‐mM Tris pH 8 buffer for protein extraction. Protein concentration was determined with 2D‐Quant kit (#80‐6483‐56; GE Healthcare), and subsequently, 10 μg of total protein (5 μl) was incubated with 2‐μl 10‐mM dithiothreitol for 30 min at room temperature to reduce disulfide bonds. Thereafter, cysteine residues were alkylated with 50‐mM 2‐chloroacetamide in 50‐mM ammonium bicarbonate (ABC) for 20 min at room temperature in the dark. Then 0.5 μg/μl of the endopeptidase LysC was added and incubated for 3 h at room temperature. Samples were then diluted 4× with 50‐mM ABC and incubated overnight at room temperature with 1‐μg trypsin/50‐μg total protein. Bond Elut C18 Omix tips (Agilent) were used to desalt and concentrate the peptide mixtures prior to LC–MS/MS measurements.

### 2.6 Liquid chromatography—tandem mass spectrometry

Measurements of each sample were performed using C18 reversed phase liquid chromatography in combination with online tandem mass spectrometry (LC–MS/MS) in data‐dependent acquisition mode. Two microliters of trypsin digested sample was loaded onto the trapping column (Acclaim PepMap 100, 75 μm × 2 cm, nanoViper, 3‐μm 100‐Å C18 particles; Thermo Fisher Scientific) using 0.1% formic acid at 7,000 nl/min for 3 min. Next, peptides were separated on a C18 reversed phase analytical column (Acclaim PepMap RSLC, 75 μm × 15 cm, nanoViper, 2‐μm 100‐Å C18 particles; Thermo Fisher Scientific) at 40 °C using a linear gradient of 5%–35% acetonitrile, 0.1% formic acid in 240 min at 600 nl/min. Measurements were performed using a nanoflow ultra‐high‐pressure liquid chromatograph (nano‐Advance; Bruker Daltonics, USA) coupled online to an orthogonal quadrupole time‐of‐flight mass spectrometer (maXis Plus; Bruker Daltonics) via an electrospray ionization source (Captive sprayer; Bruker Daltonics). The mass spectrometer was operated in positive ion mode to acquire line spectra in the mass range of *m/z* 150–2,200. Data‐dependent acquisition of MS/MS spectra (AutoMSn) was performed using a 3‐s duty cycle at 2‐Hz acquisition rate for full MS spectra and a variable number of MS/MS experiments. Precursor ions within the range of *m/z* 400–1,400 with charge state z ≥ 2+ were selected for MS/MS analysis with active exclusion enabled.

For the analysis of detected peptides of 6‐OHDA‐lesioned area versus intact area, a 120‐min gradient was used, instead of 240 min. The rest of the settings were the same as described above.

### 2.7 Database searches

Detected peptides were analyzed using MaxQuant software (v1.6.2.6) and searched against the *Rattus norvegicus* database (SwissProt) with added sequence of the known contaminant *Bos Taurus* Type I collagen using the built‐in Andromeda search engine. The decoy database used for false discovery rate validation contained the reversed protein sequences. Desired maximum false discovery rate at both peptide and protein identification levels was set to 1%. MaxQuant MS/MS database searches were performed with 10‐ppm and 0.5‐Da mass tolerance for precursor ions and fragments ions, respectively. Carbamidomethylation (at cysteine residues) was specified as fixed modification, whereas deamidation (at asparagine and glutamine residues), acetylation (protein N‐terminus), and oxidation (at methionine residues) were selected as variable modifications. Trypsin was selected as a specific enzyme, and a maximum of two missed tryptic cleavages was accepted.

For the analysis of detected peptides of 6‐OHDA‐lesioned area versus intact area, PEAKS® studio X software was using identical settings as mentioned above for MaxQuant for MS/MS database searches.

### 2.8 Gene ontology and data analysis

Gene Ontology (GO) and pathway analysis were performed using PANTHER Classification System (http://www.pantherdb.org).

Pearson's correlation between samples, principal component analysis (PCA), one‐way ANOVA with Fisher's least significant difference (LSD) post hoc test, pairwise *t*‐test with Bonferroni correction, and volcano plots of the label‐free quantification (LFQ) values were performed using metaboanalyst (https://www.metaboanalyst.ca, v4.0) and the R‐programming platform (v3.5.3, The R Foundation for Statistical Computing). An adjusted *p* value < .05 was considered as statistically significant. The threshold for the fold change (FC) in the volcano plot was set at 2.0.

## 3 RESULTS

### 3.1 Reproducibility and robustness of mass spectrometry analysis

A total of 2,219 proteins were identified using shotgun proteomics of dissected striatum samples. For 12 out of 49 samples, we observed poor mass spectrometry signals. Replicate sample preparation and measurements yielded identical results for these 12 samples that were, thus, omitted from this study because of poor sample quality (data not shown). Final number of samples in downstream data analysis can be found in Table 1.

**TABLE 1:** Number of rats per group after liquid chromatography tandem mass spectrometry

	Lesion + vehicle	Lesion + collagen	Lesion + hUC‐MSCs	Lesion + hUC‐MSCs + collagen	Intact
Day 1	4	3	4	3	3
Day 14	5	4	4	4	4

*Note.* Only good quality data were included in the downstream data analysis. Initially, each group contained five animals, except the lesion + collagen group that had *n* = 4 because one of the rats died during surgery.

**FIGURE 2:**
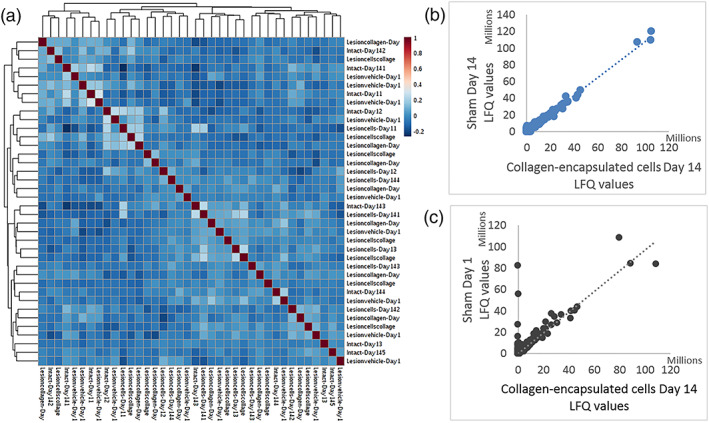
Pearson's correlations. (a) Matrix of Pearson's correlations across all samples in all treatment groups. All samples presented similar expression profiles (*r* > .88). (b) Example of the correlation of all 2,219 identified proteins individually for the highest 1‐to‐1 correlation that was observed (*r* = .99). (c) Example of the correlation of all 2,219 identified proteins individually for the lowest 1‐to‐1 correlation that was observed (*r* = .88). Blood‐derived proteins were observed in one sample only, a tissue that presented a red bloody area at dissection time and resulted in contamination with blood‐derived proteins in the lysate. LFQ, label free quantification [Colour figure can be viewed at wileyonlinelibrary.com]

To assess the robustness and reproducibility of the proteomics analysis, we calculated the coefficient of variation (CV) of the LFQ values of biological replicates. In all cases, the intra‐group CV was lower than 15% (data not shown), which is consistent with good analytical accuracy and minimal biological variation. Then, we created a matrix of Pearson's correlations of the LFQ values; all the proteins identified in one sample were correlated with all the proteins found in another one. For all comparisons, a correlation higher than .88 was obtained, indicating high robustness and reproducibility of the technique. Moreover, this high correlation factor means there is close similarity between samples, indicating similar protein expression profiles in all samples (Figure 2a,b *).* Detailed analysis of the sample with the lowest correlation coefficient (*r* = .88), which is still high (Figure 2c), revealed that this was caused by higher LFQ values of blood‐derived proteins, mainly serum albumin, hemoglobin, and murinoglobulin. Indeed, this sample showed significant visual blood contamination at dissection time. Because of power and lack of effect of those blood proteins in the rest of the proteins, this sample was included in the analysis.

### 3.2 Effects of biomaterials and hUC‐MSCs on striatal protein expression

**FIGURE 3:**
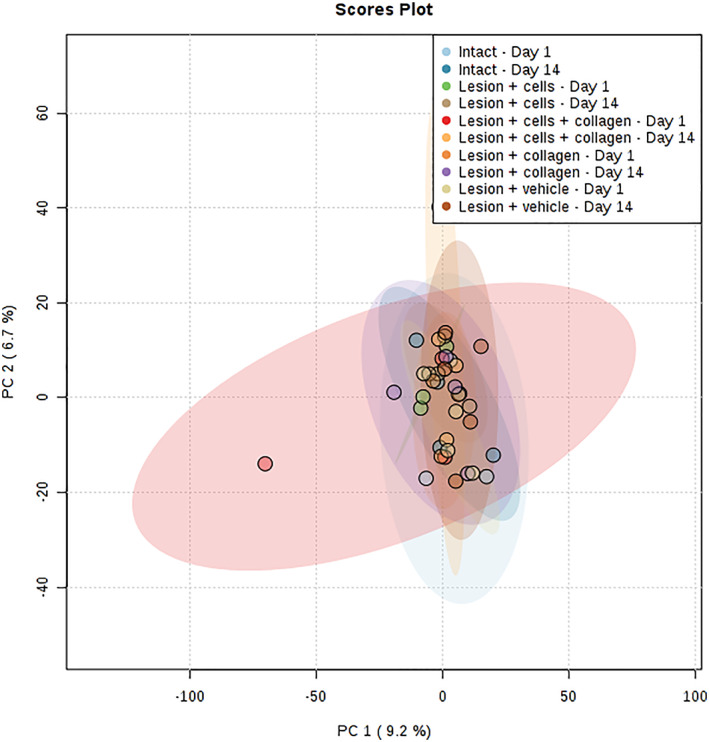
Principal component analysis of protein expression profiles of rat striatum indicating variations between intact brain and implanted brains with collagen scaffold, human umbilical cord mesenchymal stem cells (hUC‐MSCs), or collagen‐encapsulated hUC‐MSCs at either 1 or 14 days after implantation. Similar characteristics between groups can be observed. PC, principal component [Colour figure can be viewed at wileyonlinelibrary.com]

**FIGURE 4:**
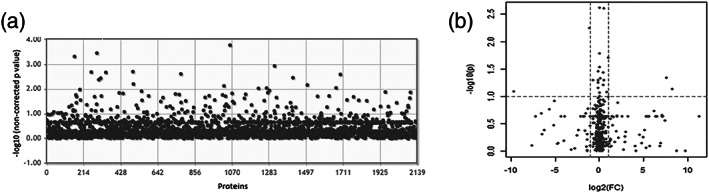
Treatment group comparisons. (a) One‐way ANOVA with post hoc test. The log 10 of the non‐corrected *p* value for all quantified proteins was plotted on the *y*‐axis and each protein at the *x*‐axis. No significant different protein levels between groups were found. The post hoc Fisher's least significant difference adjusted *p* value cutoff was .05. (b) Example of a volcano plot (intact brain Day 1 vs. implanted collagen‐encapsulated hUC‐MSC Day 1). Volcano plots connect mathematical and biological differences. At the *y*‐axis, the negative logarithm base 10 of the non‐corrected *p* value, −log 10(non‐corrected *p* value), was plotted. Threshold was set at *p* value = .05, which corresponds to a value of 1.3 after log transformation. At the *x*‐axis, the logarithm base 2 of the fold change, log2(FC), was plotted and the threshold was set at 2, which corresponds to a log2(FC) of 1. Note that the graph shows the non‐corrected *p* value and not the adjusted *p* value after post hoc test. After post hoc, no significant differences on expression profiles were observed

We compared the host tissue response in protein expression between treatment groups (intact, sham, hUC‐MSCs alone, collagen alone, or collagen‐encapsulated hUC‐MSCs) and time‐window after implantation (1 or 14 days). We analyzed large regions of striatum to map the expected dramatic changes in the brain proteome. The PCA plot showed close proximity of the scores for all samples, which shows that the majority of consistent variation in protein expression is not related to group differences that is underlined by the low percentage of variance explained by principal components PC1 (9.2%) and PC2 (6.7%) (Figure 3). One‐way ANOVA with post hoc testing was performed on the 2,219 LFQ values to explore whether there was a differential host tissue response between treatment groups. No significant difference in expression of proteins was detected (Figure 4a). We also performed pairwise comparisons of individual proteins using *t*‐tests and volcano plots to identify statistically and biologically significant changes (example shown in Figure 4b). The 23 paired group comparisons showed no differences when multiple test correction was applied (data not shown).

A GO enrichment analysis could not be performed on host tissue responses as no statistically different proteins were identified between groups. However, we did GO studies and pathway analysis of the whole protein set of identified proteins (data not shown). All identified pathways are related to brain specific processes, as well as metabolic and inflammatory processes, confirming correct protein annotation of our identified proteomes that could have been involved in the expected tissue responses to the implant.

### 3.3 Protein expression profiles between intact area and 6‐OHDA lesion area

The lack of differences between treatment conditions could be explained by a lack of treatment effect or by localization of the effect to a small region with masking of differential proteins by other proteins of the dissected area. To investigate this, we compared the proteomic profiles of the intact striatal area of the brain with the area injured with 6‐OHDA injection. We hypothesized that, if no differences between these two different areas could be detected, it would indicate that the effects were too local and, thus, could not be detected.

**FIGURE 5:**
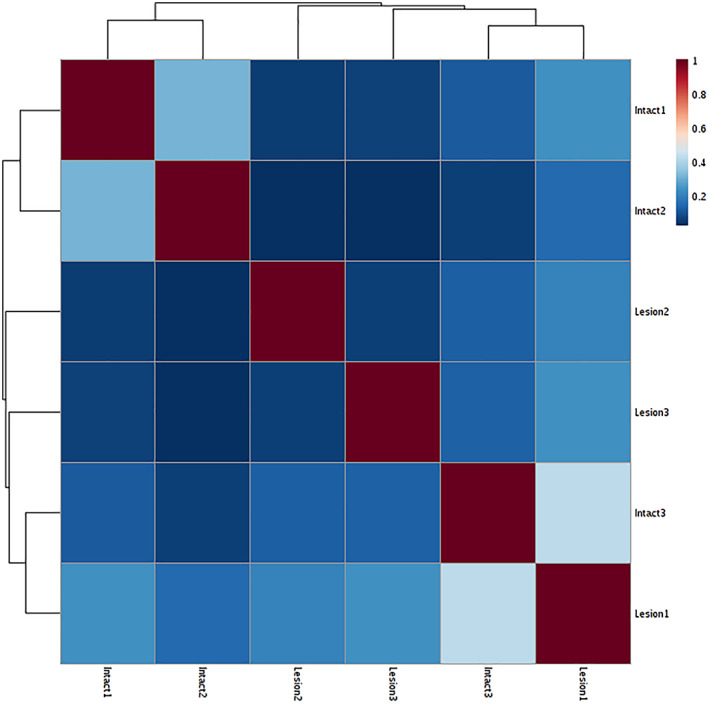
Pearson's correlation matrix between lesioned and intact area (I). High correlations between samples were observed (*r* > .89) [Colour figure can be viewed at wileyonlinelibrary.com]

**FIGURE 6:**
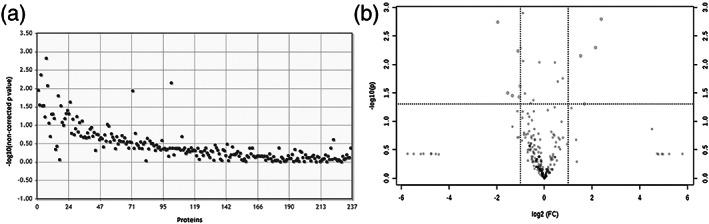
Group comparison. (a) *T*‐test with Bonferroni post hoc test. The logarithm base 10 of the non‐corrected *p* value for all identified proteins was plotted on the *y*‐axis. No significant different protein levels were found. Note that the adjusted *p* values after post hoc test are not plotted. (b) Volcano plot. On the *y*‐axis, the logarithm base 10 of the non‐corrected *p* values are plotted at a threshold of .5, corresponding to a value of 1.3 after log transformation (horizontal line). On the *x*‐axis, the logarithm base 2 of the fold change (FC) is plotted with a threshold set at 2.0, which corresponds to 1 after log transformation (vertical lines). Note that the graph shows the non‐corrected *p* value and not the adjusted *p* value after post hoc test. After post hoc, no significant differences on expression profile were observed

We analyzed four intact striatal sections of the brain hemisphere and four MFB 6‐OHDA‐lesioned ones. One intact and one lesioned area showed poor quality of the mass spectrometry signals and, thus, were not used in the downstream data analysis. A total of 551 proteins were identified in this analysis. The lower number of identified proteins is due to the shorter gradient used during the chromatography (120 vs. 240 min). Pearson's correlation between samples showed high correlation between samples (*r* > .89; Figure 5). Group comparison using *t*‐test as well as volcano plot analysis with post hoc testing showed no significant differences between groups (Figure 6), indicating that the expected effects are too local to be detected.

### 3.4 Viability of hUC‐MSCs into the collagen hydrogel

Encapsulation and implantation of 3 μl of stem cells (10,000 cells/μl) was previously described with positive outcomes: diminished the host brain's response and increased survival and reinnervation of the implanted cells (Hoban et al., 2013; Moriarty et al., 2017; Moriarty et al., 2019). However, in none of these studies, hUC‐MSCs were used. Therefore, we considered the possibility that hUC‐MSCs survival is limited by collagen encapsulation at the concentrations described, and as a result, no effects in our study could be observed. We observed that the viability of high concentrated cells (10,000 cells/μl) dropped to 22.3 ± 31.6% upon encapsulation, whereas the viability of low concentrated cells (2,500 cells/μl) was around 67.6 ± 9.7%, similar to the viability obtained in 2D cultures (68.1 ± 14.1). The results suggest that the cells might not have survived long time inside the hydrogel after implantation into the rat's striatum and, thus, caused non‐effect on the host proteome.

## 4 DISCUSSION

The present study is an explorative assessment to the host proteomic changes following implantation of collagen‐encapsulated hUC‐MSCs into the striatum of the 6‐OHDA rat model of PD by using LC–MS/MS. Results showed high robustness and reproducibility of mass spectrometry analyses with low variation between biological replicates. Group comparison between treatment conditions and time post‐treatment showed no significant differences in protein expression levels between groups. This result was unexpected as we hypothesized that collagen‐encapsulated hUC‐MSCs would produce widespread changes in the host tissue. Based on previous studies (Hoban et al., 2013), we expected that 24 h after implantation bovine collagen and increased levels of inflammation‐related proteins would be detected, which would have normalized after 14 days, a period during which the level of trophic factors and other proteins involved in biological processes would have changed. Previous similar studies on 6‐OHDA rat models implanted with collagen‐encapsulated ventral mesencephalic (VM) cells in combination with glial cell line‐derived neurotrophic factor (GDNF) loaded into the hydrogel observed improvement of rat behavior, reduction of microgliosis and astrogliosis, and an increase in GDNF (Moriarty et al., 2017; Moriarty et al., 2019). This is in contrast to the findings in our study and might be due to the use of hUC‐MSCs instead of VM cells, the lack of supportive GDNF and/or the analytical approach. We used high‐throughput mass spectrometry for proteomic profiling to get a broad impression of proteomic changes, whereas others used immunohistochemistry to analyze specific markers of microgliosis, astrogliosis, and GDNF.

We used hUC‐MSCs instead of another type of stem cell because hUC‐MSCs are largely available, easy to obtain and to expand, raise fewer ethical concerns, are non‐immunogenic, and release several anti‐inflammatory proteins and key neurotrophic factors. Specifically, hUC‐MSCs secrete large amounts of GDNF, which is specifically beneficial for dopaminergic neurons (El Omar et al., 2014; Hass et al., 2011). hUC‐MSCs also present benefits in comparison with bone marrow MSCs (BM‐MSCs): hUC‐MSCs weakly express HLA‐ABC (less immunogenic), grow faster because of the younger origin, and have a higher differentiation potential (El Omar et al., 2014; Hass et al., 2011). Some scientists in the field consider hUC‐MSCs not to be good candidates for brain regenerative therapy because of their limited potential to differentiate into neurons (Barker, Drouin‐Ouellet, & Parmar, 2015; Stoker & Barker, 2018). However, preclinical studies with cell models and rat models of PD yielded beneficial effects when using hUC‐MSCs as a treatment for PD (Liu et al., 2014; Mathieu, Roca, Gamba, Del Pozo, & Pitossi, 2012; Xiong et al., 2010), probably because of their valuable secretome (El Omar et al., 2014; Teixeira et al., 2017). To date, there is one ongoing clinical trial in humans using hUC‐MSCs in PD patients (NCT03550183; https://clinicaltrials.gov/).

The encapsulation and implantation procedures that we followed were previously described with positive outcomes (Hoban et al., 2013; Moriarty et al., 2017; Moriarty et al., 2019). However, there were two main differences. First, hUC‐MSCs have not been used before, but rat dopamine‐derived VM cells or rat BM‐MSCs were used, which showed good cell viability. In these studies, cell viability was assessed 1 day after encapsulation. It might be possible that survival is cell‐type dependent or that hUC‐MSCs survival overtime is more limited by collagen encapsulation than previously used stem cell types. To test this hypothesis, we performed an in vitro study, in which two different concentrations of cells were encapsulated into collagen hydrogel for 5 days. We observed that viability of encapsulated cells decreased with higher concentration of cells. Thus, viability of the cells might have influenced our results. However, more detailed cell survival analysis should be performed to provide a more definite answer. Second, in previous experiments, GDNF‐overexpressing cells were used, or the scaffold was loaded with GDNF and cells. The elevated presence of GDNF yielded a higher reinnervation, better behavior outcome, and lower immune response in comparison with the encapsulated cells without GDNF. The protective and supportive role of GDNF for dopaminergic neurons has already been reported in several studies (Meka et al., 2015; Yasuhara et al., 2017). Thus, probably hUC‐MSCs should overexpress or be co‐encapsulated with GDNF to potentiate their therapeutic effects, which is supported by the positive outcome of a study in which hUC‐MSCs overexpressing hepatocyte growth factor (HGF), a survival factor for motor neurons, were used to treat a cell model of PD (Liu et al., 2014). In this study, we did not load the  scaffolds with GDNF because we thought that the normal expression of GDNF by hUC‐MSC would be enough.

However, we believe that the main two explanations for the lack of differences in protein expression levels in the present study are the following: (a) there may be no effects strong enough to be detected using shotgun proteomics and (b) the relative large dissected area in comparison with the small area of implantation diluted the effects. This second explanation is corroborated by the finding that also no differences were observed between intact area (no surgery) and lesion area (6‐OHDA injection). Surgical procedures activate the immune response and cause changes in the surrounding area, and 6‐OHDA injection causes the death of dopaminergic neurons. In LC–MS/MS, the detection of low‐abundant proteins is hampered by the presence of high‐abundant proteins (Qian, Jacobs, Liu, Camp, & Smith, 2006). Consequently, common and abundant proteins from the cell membrane, extracellular matrix, and organelles have high probability to be detected and to mask the low‐abundant proteins, such as trophic factors, cytokines, and chemokines. This masking effect might be intensified by the local effects of the transplant, which affects a relatively small area of the brain in comparison with the rest of the hemisphere. Immunohistochemistry studies showed that transplantation may have a radial effect extending up to 200–250 μm away from the site of injection (Moriarty et al., 2017). Also, literature search revealed that similar studies using shotgun mass spectrometry in rat brains focused on larger areas of interest with bigger effects, pooled several brains, and/or coupled mass spectrometry to a previous microdissection of the tissue or protein separation techniques (Campos‐Martorell et al., 2014; Gellen et al., 2017; Quanico, Franck, Wisztorski, Salzet, & Fournier, 2017; Zhang et al., 2018).

Given the lack of widespread differences of protein expression levels, other techniques may be better suited to unravel the proteomic changes after implantation. Laser capture microdissection (LCM) in combination with LC–MS/MS or mass spectrometry imaging (MSI) may be alternatives. Both techniques present advantages but also limitations. LCM has already been used to isolate brain cell populations for gene and protein expression analysis (Fustin, Karakawa, & Okamura, 2017; Sussulini & Becker, 2015). However, in our experimental setting, we would need to dissect a donut‐shaped tissue area to be able to differentiate the host tissue response from the transplanted cells, which would be complicated and time consuming. MSI uses the combination of molecular mass analysis and spatial information, providing visualization of molecules directly from a complex tissue surface. MSI has become a workhorse for proteomic and peptidomic studies (Huber et al., 2018; Norris & Caprioli, 2013). However, MSI presents some drawbacks: the spatial resolution is lost due to on slide tryptic digestion, and the proteome coverage is limited to a few hundred proteins. Nonetheless, promising advances have been made to study the proteome of murine brains (Heijs et al., 2015; Huber et al., 2018; Stoeckli, Staab, Staufenbiel, Wiederhold, & Signor, 2002).

In summary, we intended to determine the proteomic changes in the striatum of the 6‐OHDA PD rat model after implantation of collagen hydrogel‐encapsulated hUC‐MSCs. Our results confirmed the high robustness and reproducibility of our analysis but could not determine differences between groups. We believe that the lack of differences between groups is because, if there is an effect, it is neither strong nor widespread. For future experiments, we suggest that LCM combined with LC–MS/MS or MALDI imaging techniques, despite their own disadvantages, may yield a more detailed insight into the host tissue proteomic response to the transplanted encapsulated cells in order to be able to assess the efficacy and safety of collagen‐encapsulated hUC‐MSCs as a disease modifying therapy for PD.

## CONFLICT OF INTEREST

The authors have declared that there is no conflict of interest.

## AUTHOR CONTRIBUTIONS

A.S. collected the data, performed the data analysis, and wrote the manuscript. S.C. and V.A. performed the in vivo experiments. P.K. helped with the biostatistical analysis. A.S., H.W., J.G., H.B.K., and M.V. interpreted the data and revised the manuscript. All authors read the manuscript for intellectual content and commented on the final version of the manuscript.
